# From Levulinic Acid to Imines: Creating Biobased, Recyclable, Cross-linked Rubbers through Covalent Adaptive Networks

**DOI:** 10.1021/acspolymersau.5c00108

**Published:** 2025-09-26

**Authors:** Luca Lenzi, Juan Carlos Chicharro, Micaela Degli Esposti, Davide Morselli, Marianella Hernández Santana, Paola Fabbri

**Affiliations:** † Department of Civil, Chemical, Environmental and Materials Engineering (DICAM), Università di Bologna, Via Terracini 28, 40131 Bologna, Italy; ‡ National Interuniversity Consortium of Materials Science and Technology (INSTM), Via Giusti 9, 50121 Firenze, Italy; § Institute of Polymer Science and Technology (ICTP) CSIC, Juan de La Cierva 3, 28006 Madrid, Spain

**Keywords:** covalent adaptive network, rubber, imine bonds, hydrogen bonds, recycling, levulinic acid, biobased

## Abstract

Dynamic covalent bonds provide a powerful tool to design recyclable rubber-based materials. Among possible strategies, imine chemistry offers a valuable approach for achieving an adaptive network. In this work, for the first time, the use of biobased ketones and amines as cross-linkers in a rubber network is used. Specifically, epoxidized natural rubber (ENR) was cross-linked with glycerol trilevulinate (GT) and hexamethylene diamine (HMDA) to produce a fully biobased and recyclable hybrid network based on imine and hydrogen bonds. Comprehensive characterization confirmed the formation of a hybrid adaptive network, while mechanical tests demonstrated that the optimal formulation (5 phr GT and 5 phr HMDA) achieved the best performance after recycling, showing a significant increase in tensile strength while maintaining stable strain at break. The material’s ability to reconstruct its network upon reprocessing was supported by cross-link density measurements via swelling and mechanical analyses, while dielectric investigations further confirmed the presence of dynamic interactions. The observed recyclability was thus attributed to the synergistic effect of covalent and noncovalent bonds, which reorganized effectively to preserve network integrity and mechanical performance. This work demonstrates the potential production of a biobased, recyclable and adaptable rubber network with excellent mechanical properties, highlighting how levulinic acid derivatives represent an optimal system for the development of sustainable rubber materials.

## Graphical abstract

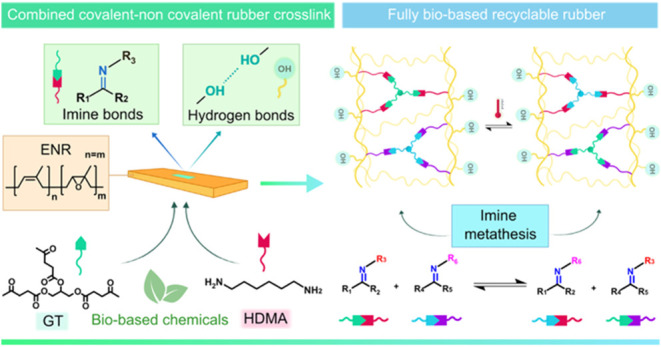

## Introduction

The demand for rubber products continues to grow, driven by their unique properties, such as elasticity, resilience, and durability. These characteristics make rubber indispensable for applications requiring flexibility and strength, including tires, medical devices, and industrial components.^1,2^ However, this growth poses significant challenges, particularly in terms of environmental sustainability and waste management.^3,4^ Natural rubber production, sourced primarily from *Hevea brasiliensis*, is vulnerable to climate fluctuations and land-use constraints, while the end-of-life management of the derived rubber products remains a critical issue, with limited recyclability and persistent waste accumulation.^5^ Additionally, substances used in conventional rubber cross-linking (also called vulcanization), such as sulfur and other additives,^6^ can pose risks to human health and the environment.^7^ While these agents create strong, durable covalent networks that enhance mechanical properties of the material, they also make rubber difficult to degrade or recycle efficiently.^8–10^ Current recycling methods, including pyrolysis and cryogenic grinding, are energy-intensive and produce low-quality materials with limited applications.^11,12^ As result, a significant portion of rubber waste, including millions of tires, ends up in landfills or incinerated, contributing to environmental pollution and the loss of valuable resources.

This situation has generated an urgent need for innovative approaches to address the environmental and economic challenges associated with rubber production and disposal.^13^ Covalent adaptive networks (CANs) have emerged as a promising solution, enabling the creation of reprocessable materials and intrinsically recyclable systems.^14^ These networks rely on reversible covalent bonds that can break and reform under specific conditions, such as heat or chemical treatment, allowing for efficient recycling while preserving the material’s mechanical integrity through recycling cycles.^15^ Among these, imine bonds have gained particular attention for their application in rubber materials, demonstrating remarkable reprocessability and recyclability.^16–20^ These bonds can undergo exchange reactions under mild conditions,^21^ making them especially attractive for the fabrication of recyclable and reconfigurable polymer systems. Imine bonds are typically formed through the condensation of aldehydes or ketones with primary amines, offering a modular platform for dynamic network construction.^22^ Despite their potential, the vast majority of imine-based CANs still rely on fossil-derived, toxic, or environmentally persistent reagents, posing limitations in terms of sustainability and scalability.^23,24^ In response, vanillin and other biobased aldehydes have emerged as promising building blocks for the design of sustainable, recyclable polymer networks.^25^

On the contrary, the use of biobased ketones in dynamic covalent networks remains largely unexplored, despite the promising potential of platform molecules such as levulinic acid (LA),^26^ which is expected to be produced on a large scale with the advancement of the biorefinery sector.^27^ LA already showed encouraging results in the production of reprocessable thermoset materials. Specifically, Huang et al.^28^ demonstrated the potential of ketone–amine chemistry for cross-linking lignin via dynamic imine formation, underscoring the viability of this route in biomass-derived systems. Similarly, Liu et al.^29^ showed that LA-epoxy monomers could be used for producing thermosets with dynamic imine bonds, enabling reprocessability. Despite these advances, the potential of LA and its derivatives remains scarcely investigated, especially as potential cross-linkers for the production of adaptable networks in rubber systems.

In order to further explore the use of biobased ketones for imine-based rubbers, we propose the use of glycerol trilevulinate (GT), a biobased molecule derived from LA and glycerol, in combination with hexamethylene diamine (HMDA), to establish a fully biobased dynamic cross-linking network within epoxidized natural rubber (ENR). GT, which has previously demonstrated excellent plasticizing properties,^30,31^ offers significant potential in forming imine bonds through ketone groups,^22^ while HMDA, traditionally produced from fossil resources, can now be obtained through environmentally friendly processes.^32–34^ Moreover, to the best of our knowledge, this is the first study to employ ketones as precursors for dynamic imine-based cross-linking in rubber formulations, offering a sustainable approach to design recyclable elastomers.^35^ The combination of GT and HMDA allows the creation of a hybrid cross-linked network within ENR, where reversible imine bonds ensure recyclability, while permanent covalent bonds formed between HMDA and ENR’s epoxy groups anchor the rubber chains forming a cross-linked network. Additionally, hydrogen bonds, formed from the epoxy ring-opening reaction, complement the reversible covalent bonds, making the system a hybrid that combines both covalent and noncovalent interactions for enhanced stability and adaptability.^36^ This hybrid bonding system not only enhances the material’s mechanical performance and recyclability but also embodies the principles of a circular economy by utilizing renewable resources and enabling repeated use addressing the growing demand for sustainable, high-performance rubber systems while significantly reducing their environmental impact.

## Experimental Section

### Materials

Epoxidized natural rubber (ENR) with 50% of epoxidation was provided by Tun Abdul Razak Research Centre (TARRC) of the Malaysian Rubber Board, with a declared glass transition temperature (*T*_g_) of – 24 °C, and density of approximately 0.92 g cm^–3^. Hexamethylene diamine (HMDA, purity ≥ 98%) was purchased from Sigma-Aldrich and used without further purification. Glycerol trilevulinate (GT) was synthesized according to the procedure described in detail elsewhere.^30,31^ Briefly, glycerol (GLY) was reacted with an excess of LA in a solvent-free catalyzed esterification, with water continuously removed to drive the reaction toward the desired product. GT was then isolated by solvent extraction, yielding a yellowish liquid with 85% yield.

### Preparation of Compounds

To design rubber formulations with balanced properties and recyclability, four formulations were prepared as reported in Table 1. Specifically, the effect of GT on the dynamic cross-linking system was investigated by varying its concentration, in order to evaluate how the balance between epoxy–amine and ketone–amine reactions influenced the resulting network. Formulation H5, containing only HMDA (5 phr) as cross-linking agent, was used as reference to assess the impact of GT and imine bonds on the system’s recyclability. The content of 5 phr of HMDA was selected to ensure the right balance between cross-link density and molecular mobility, a key factor for enabling reversibility and efficient recycling. The other formulations, H5GT5, H5GT7.5, and H5GT10, were produced incorporating 5, 7.5, and 10 phr of GT, respectively, while maintaining a constant HMDA content of 5 phr. The H5GT5 formulation was produced based on theoretical calculations, assuming that half of HMDA’s amino groups react with the epoxy rings of ENR, while the other half bonds with the ketone groups of GT, ensuring the reaction of all available ketone moieties in the cross-linking process. In order to evaluate whether the theoretical amount of GT was sufficient to achieve the desired reversible network, additional formulations with higher GT content (H5GT7.5 and H5GT10) were prepared. The rubber compounds were prepared using an internal mixer (Haake Rheomix 9000) with Banbury-type rotors, mixing the matrix and additives for 20 min at room temperature and a constant speed of 60 rpm.

**Table 1:** ENR Formulations with Increasing GT Content Expressed in Parts Per Hundred Rubber (phr)

materials	H5	H5GT5	H5GT7.5	H5GT10
ENR (phr)	100	100	100	100
HMDA (phr)	5	5	5	5
GT (phr)	0	5	7.5	10

### Processing Conditions and Sample Preparation

Differential scanning calorimetry (DSC, 214 Polyma, Netzsch) was used to determine the optimal duration of the heat treatment to induce the cross-linking. Isothermal DSC provided valuable insights on the curing process by tracking the exothermic reaction associated with cross-linking. The analysis was conducted at 180 °C to determine the time required to achieve a fully cross-linked material. The exothermic peak in the DSC curve was directly related to the extent of cross-linking, as the heat released during this reaction was proportional to the degree of bond formation. By examining the onset, peak, and end point of the exothermic reaction, the curing time needed for a stable cross-linked network was estimated.^37^ Figure S1 shows the optimal curing time (*t*_curing_) required to achieve the cross-linked state for the reference compound H5. The other compounds (H5GT5, H5GT7.5, and H5GT10) were processed keeping the same *t*_curing_ of H5.

All samples were subsequently vulcanized using a hydraulic press at 180 °C and 200 bar for the duration of the *t*_curing_. Approximatively 20 g of each compound were molded into rectangular sheets with a thickness of 2 mm and a length of 110 mm. Following vulcanization, the samples were cooled for additional 5 min before characterization.

### Fourier Transform Infrared Spectroscopy (FTIR)

FTIR analysis was performed to study the vulcanization reaction, as well as to characterize the materials after the recycling process, using a PerkinElmer Spectrum Two spectrometer equipped with a diamond ATR (attenuated total reflection) crystal. The spectra were recorded over a range of 4000 to 400 cm^–1^, with 16 scans collected each sample. The data were analyzed using Spectrum 10 software (PerkinElmer).

### Recycling Procedure

Recycling was carried out by first cutting the virgin cross-linked rubber samples into smaller pieces, which were subsequently ground into a fine powder using a cryogenic ball mill (Retsch Cryomill) for 40 min. The powder was subsequently processed in a two-roll mill (MGN-300S, Comerio Ercole) for 2 min to form a uniform sheet, with water circulation system employed to prevent excessive heat build-up and avoid cross-linking during processing. Finally, the material was recross-linked in a hot press at 180 °C and 200 bar for 2 h.

### Scanning Electron Microscopy (SEM)

The morphology of the virgin and recycled compounds was examined using a scanning electron microscope (SEM) (JSM 3690, JEOL). The cross-section of each sample was prepared by cryo-fracturing rectangular-shaped specimens in liquid nitrogen. The so-obtained cross sections were then coated with gold (approximately 10 nm) by an electrodeposition method to impart electrical conductivity. The samples were analyzed applying an accelerating voltage of 25 kV.

### Swelling Test

Swelling tests were performed on five square specimens from each vulcanized compound, using toluene as the solvent.^38^ The initial mass of each specimen was measured in air, then samples were immersed in toluene for 72 h. Afterward, samples were removed from toluene, weighed, and reweighed after the solvent was evaporated. The cross-link density (*ρ_crosslink_*), expressed in moles per volume of rubber (mol·cm^–3^), was determined using the Flory–Rehner equation^39,40^ following reported as eq 1

1 $$\rho_{\mathrm{crosslink}}=\rho_{r}/2M_{c}$$

where *ρ_r_* is the density of the compound and *M*_c_ is the molecular weight between cross-links.

### Thermal Characterization

DSC investigations were performed under a nitrogen atmosphere to evaluate potential structural changes in the compounds after recycling. The analysis involved heating both virgin and recycled samples from −80 to 200 °C at 20 °C·min^–1^ to measure the glass transition temperature (*T*_g_).

### Broadband Dielectric Spectroscopy (BDS)

Dielectric permittivity measurements were carried out using a high-resolution dielectric analyzer (α, Novocontrol) equipped with a temperature control system (QUATRO, Novocontrol). The measurements spanned a frequency range from 10^–1^ to 10^6^ Hz and a temperature range from −100 to 100 °C (5 °C of increment). Virgin and recycled samples were placed between two gold-plated electrodes. The complex dielectric function ε*­(ω) was calculated with eq 2

2 $$\varepsilon^{*}\left(\omega \right)=\varepsilon^{\prime }\left(\omega \right)-i\varepsilon^{\prime \prime }\left(\omega \right)$$

where ε′(ω) is the real part and is proportional to the energy stored reversibly in the system and ε″(ω) is the imaginary part, proportional to the dissipated energy (also known as dielectric loss).^41^

The dielectric relaxation processes were analyzed quantitatively by fitting the frequency spectra to the Havriliak–Negami (HN) function^42^ given by eq 3

3 $$\varepsilon_{\mathrm{HN}}^{*}\left(\omega \right)=\varepsilon_{\infty }+{\Delta \varepsilon /\left[1+{\left(i\omega {\cdot \tau }_{\mathrm{HN}}\right)}^{b}\right]}^{c}$$

where τ_HN_ represents the characteristic HN relaxation time, which corresponds to the most probable relaxation time from the relaxation time distribution function. ω is the angular frequency (ω = 2π·*f*), Δ*ε* is the dielectric strength of the relaxation which is related to the area under the absorption curve given by (Δε = ε_s_ – ε_∞_), where ε_∞_ and ε_s_ are the unrelaxed and relaxed values of the dielectric constant, respectively. *ε*_HN_^*^ *(ω)* is the frequency-dependent HN complex dielectric permittivity, and *b* and *c* are shape parameters (0 < *b*; *b·c* ≤ 1) that characterize the symmetric and asymmetric broadening of the relaxation time distribution, respectively.

The HN relaxation time τ_HN_ is related to the frequency of maximum loss (*f*_max_) by eq 4

4 $$\tau_{\max}=1/2\pi \cdot f_{\max}=\tau_{\mathrm{HN}}\cdot {\left[\sin\left(b\cdot \pi /2+2c\right)\right]}^{-1/b}{\cdot \left[\sin\left(b\cdot c\cdot \pi /2+2c\right)\right]}^{1/b}$$

The temperature dependence of the dielectric relaxations can be adjusted using the Vogel–Fulcher–Tammann–Hesse (VFTH) function,^43^ according to eq 5

5 $$\tau_{\max}=\tau_{0}\cdot \exp\left(B/T-T_{0}\right)$$

where *B* and τ_0_ are empirical parameters, and *T*_0_ is the Vogel temperature.

### Tensile Tests

The mechanical properties of the vulcanized rubber compounds were evaluated using a universal testing machine (Instron 4204) on dog bone-shaped specimens, with a gauge length of 20 mm and the thickness of 2 mm, following the standard method ASTM D638 (2022). The tests were conducted with a crosshead speed of 500 mm·min^–1^ and a 1 kN load cell. Modulus at 100% of strain (*M*_100_), tensile strength (σ_break_) and elongation at break (ε_break_) were extrapolated from the stress–strain curves of the virgin and recycled formulations. Three samples from each compound were tested, and the average values, along with their standard deviations were calculated. The cross-link density (ν_crosslink_) was also estimated using the Mooney–Rivlin model, which relates the mechanical response under uniaxial tension to the network structure.^44,45^ Stress–strain data obtained from tensile testing were used to calculate the reduced stress (σ_red_), which was then plotted against the reciprocal of the stretch ratio (λ^*–1*^), where λ = 1 + ε and ε is the strain recorded with tensile test. The relationship is described by eq 6

6 $$\sigma_{\mathrm{red}}=\sigma /\left(\lambda -\lambda^{-2}\right)={2C}_{1}+{2C}_{2}\cdot \lambda^{-1}$$

where, *C*_1_ reflects the contribution from cross-linking units, while *C*_2_ is associated with physical entanglements, also known as Mooney–Rivlin elastic constant. From the linear portion of the plot, the y-intercept corresponds to *2C*_1_, and the slope to *2C*_2_.

The values of *C*_1_ were then used to determine ν_crosslink_ of the virgin and recycled compounds using eq 7

7 $$\nu_{\mathrm{crosslink}}={2C}_{1}/\left(N_{a}\cdot k\cdot T\right)$$

where *N*_a_ is Avogadro’s number (6.022 × 10^23^ mol^–1^), *k* is the Boltzmann constant (1.38 × 10^–23^J·K^–1^), and *T* is the testing temperature in kelvin (K). The resulting ν_crosslink_ was subsequently expressed in mol·cm^–3^.

## Results and Discussion

### Covalent Adaptive Network Formation Mechanism

Dynamic covalent chemistry offers an effective route to design elastomeric networks capable of reprocessing, addressing key limitations of traditional cross-linked rubbers. In this context, ENR is highly reactive compared to natural rubber, due to the presence of epoxy groups. These functional sites enable the formation of covalent bonds with various cross-linking agents^46–48^ including HMDA.^49,50^ When HMDA reacts with the epoxy groups in ENR, strong covalent bonds are established between the diamine and rubber chains, creating a durable cross-linked network tailored to provide the desired mechanical response. Additionally, the opening of the oxirane rings generate hydroxyl groups in the rubber chains, which can potentially form hydrogen bonds, thereby enhancing network stability and support recycling or repair.^51–53^ Figure 1A illustrates the reaction mechanism between ENR and HMDA, where covalent and hydrogen bonding contribute to the formation of the cross-linked network. In this work, the third key component for establishing a covalent adaptable network with ENR was GT which, beyond its plasticizing properties,^30,31^ also represents an interesting molecule for formation of reversible bonds. In fact, the presence of three ketone groups within GT structure accounts for its reactivity, which can undergo condensation with primary amino groups, such as those present in HMDA (Figure 1B). As illustrated in Figure 1C, the reaction between ketones and primary amines leads to the formation of imine bonds,^22^ also known as Schiff bases, which are covalent yet reversible, thus possibly enabling dynamic cross-linking within the rubber.^21^

**Figure 1:**
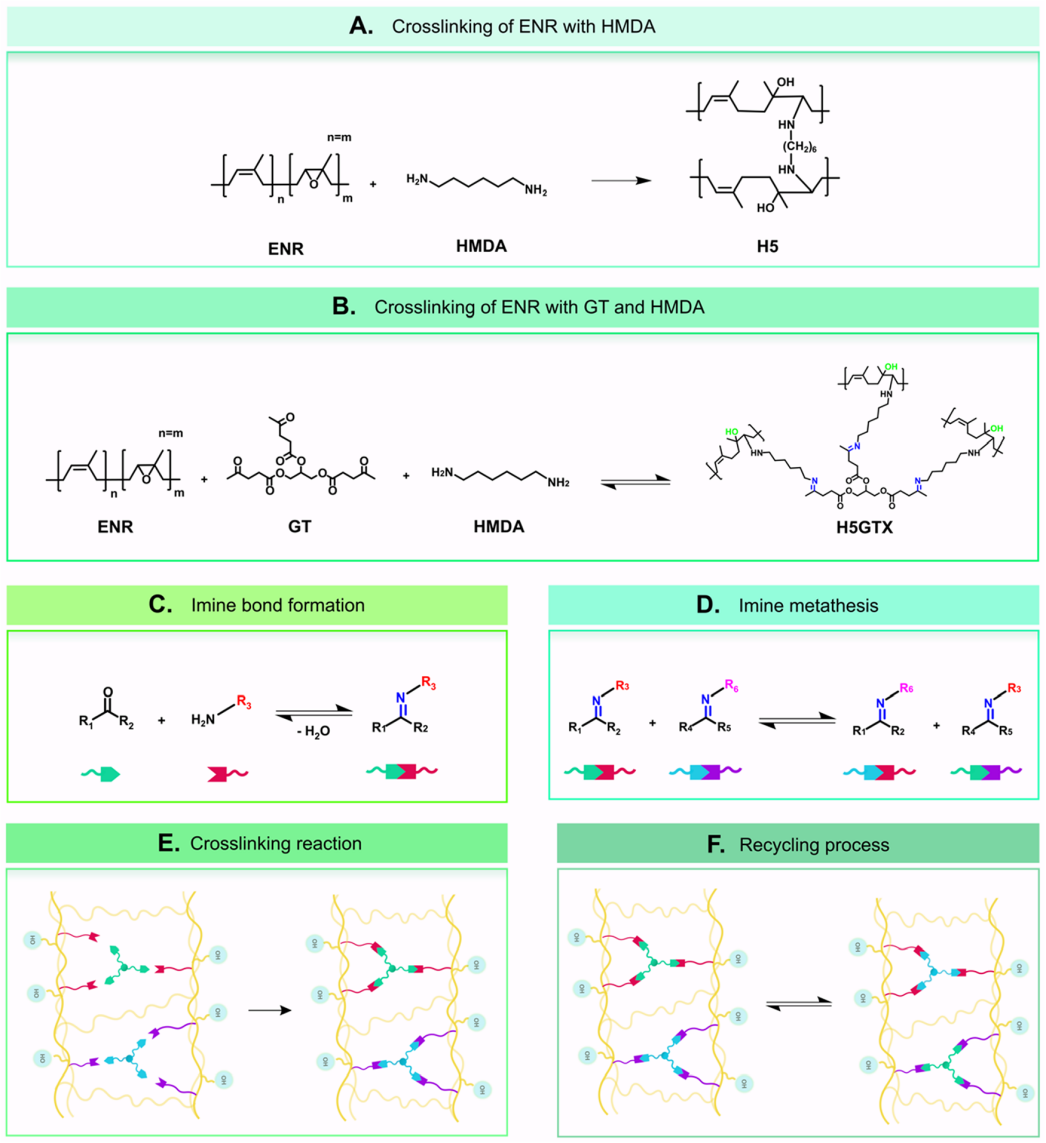
(A) Reaction scheme between ENR and HMDA. (B) Reaction scheme of the cross-linking between ENR, HMDA and GT. (C) Reaction scheme between ketone and primary amine for imine formation. (D) Imine metathesis reaction scheme. (E) Graphical representation of the cross-linking reaction. (F) Graphical representation of the recycling process enabled by imine metathesis and hydrogen bonding.

A key feature of imine-based networks is their ability to undergo imine metathesis, allowing continuous bond rearrangement when heated. This process, shown in Figure 1D, facilitates the reconfiguration of the network, allowing its recyclability as in the work of Liu et al.,^54^ who demonstrated that imine metathesis can facilitate the stress relaxation of polymer networks, improving their reprocessability during heating treatments. In addition to covalent bonds, the system proposed in this work also benefits from hydrogen bonding involving hydroxyl groups formed during the cross-linking process, which contribute to network stabilization and promote adaptability during reprocessing.^16^ After the initial cross-linking process and the formation of imine bonds (illustrated in Figure 1E), the system can undergo recovery and reprocessing upon heating. The hybrid network, combining both covalent (imine) and noncovalent (hydrogen) interactions, responds to heat through imine metathesis, allowing for controlled reprocessing and recyclability, as represented in Figure 1F.

FTIR spectroscopy was employed to compare the chemical structures of the unvulcanized (UV) and vulcanized (V) rubber and to investigate whether the combined cross-link network reported in Figure 1B was effectively formed. As shown in Figure 2A, GT exhibits a characteristic peak at 1733 cm^–1^ due to the stretching of carboxylic esters, along with another peak at 1713 cm^–1^ corresponding to ketone groups. These peaks were also observed in the UV samples, as depicted in H5GT5 (UV) spectrum in Figure 2B, thus confirming the successful incorporation of GT into the ENR matrix. After vulcanization, H5GT5 (V) no longer exhibited the ketone peak at 1713 cm^–1^, while the ester peak (1733 cm^–1^) remains broader but detectable, as shown in the inset of Figure 2C. Furthermore, H5GT5 (V) showed the appearance of a distinct peak at 1660 cm^–1^, attributed to the C = N stretching vibration, confirming the formation of imine bonds.^55,56^ The detection of a weaker peak at the same wavelength for H5GT5 (UV) sample (Figure 2C) may have been originated from partial ketone–amine condensation triggered during internal mixing. The combination of shear forces and localized frictional heating is known to activate chemical reactions in elastomer, as shown in mechanochemistry studies of butadiene rubber, likely inducing early imine bond formation in the system before the curing process.^57^ Hydroxyl groups were also identified by the characteristic broad peak at the wavenumber range of 3600–3200 cm^–1^, corresponding to the O–H stretching vibrations, indicating that epoxy rings of ENR reacted covalently with some amino functionalities of HMDA, as illustrated in Figure 1B. Spectra before and after vulcanization of the reference sample (H5), shown in Figure S2A, also confirm the presence of the hydroxyl groups vibration at 3600–3200 cm^–1^, proving that ENR reacted with HMDA following the reaction path presented in Figure 1A. The spectra of H5GT7.5 and H5GT10, reported in Figure S2B,C respectively, presented the same features of the combined cross-link network here reported for H5GT5. The impact of GT content on the morphology of the rubber compounds was evaluated by SEM. High-magnification images of the vulcanized samples are presented in Figure 2D, while additional low-magnification views are provided in Figure S3. As shown in Figure 2D, H5 and H5GT5 exhibited smooth and homogeneous surfaces, indicating efficient vulcanization process. In contrast, H5GT7.5 revealed the presence of a secondary phase, appearing as brighter a region, which becomes even more pronounced in H5GT10 (the formulation with the highest GT content). This phenomenon was likely caused by the excess GT incorporated into the system, which reacted with HMDA’s amino groups, disrupting the equilibrium and promoting the formation of a network primarily composed of GT-HMDA imine linkages. This secondary phase, with a predominant polyimine structure, consisted of multiple GT and HMDA units bonded together, thereby depleting amino functionalities that would otherwise react with ENR’s epoxy groups. The proposed mechanism for this side reaction and the resulting chemical structure are illustrated in Figure S4. Additional evidence of the side-reaction, occurring when GT content exceeds the stoichiometric ratio, was provided by the comparison of the FTIR spectra of the vulcanized samples shown in Figure 2E. In the spectral region characteristic of hydroxyl groups (3600–3200 cm^–1^), the signals corresponding to these functionalities in H5GT7.5 and H5GT10 were noticeably weaker than in H5GT5. This reduction in intensity supports the hypothesis of secondary phase formation. As shown in the possible mechanism of the side reaction illustrated in Figure S4, the excess of GT likely consumed a substantial portion of the amino groups, thereby reducing their availability to react with ENR’s epoxy groups and consequently limiting the generation of hydroxyl groups within the system. The formation of this secondary phase is schematically depicted in Figure 2F and visible in optical photographs of the vulcanized compounds in Figure 2G.

**Figure 2:**
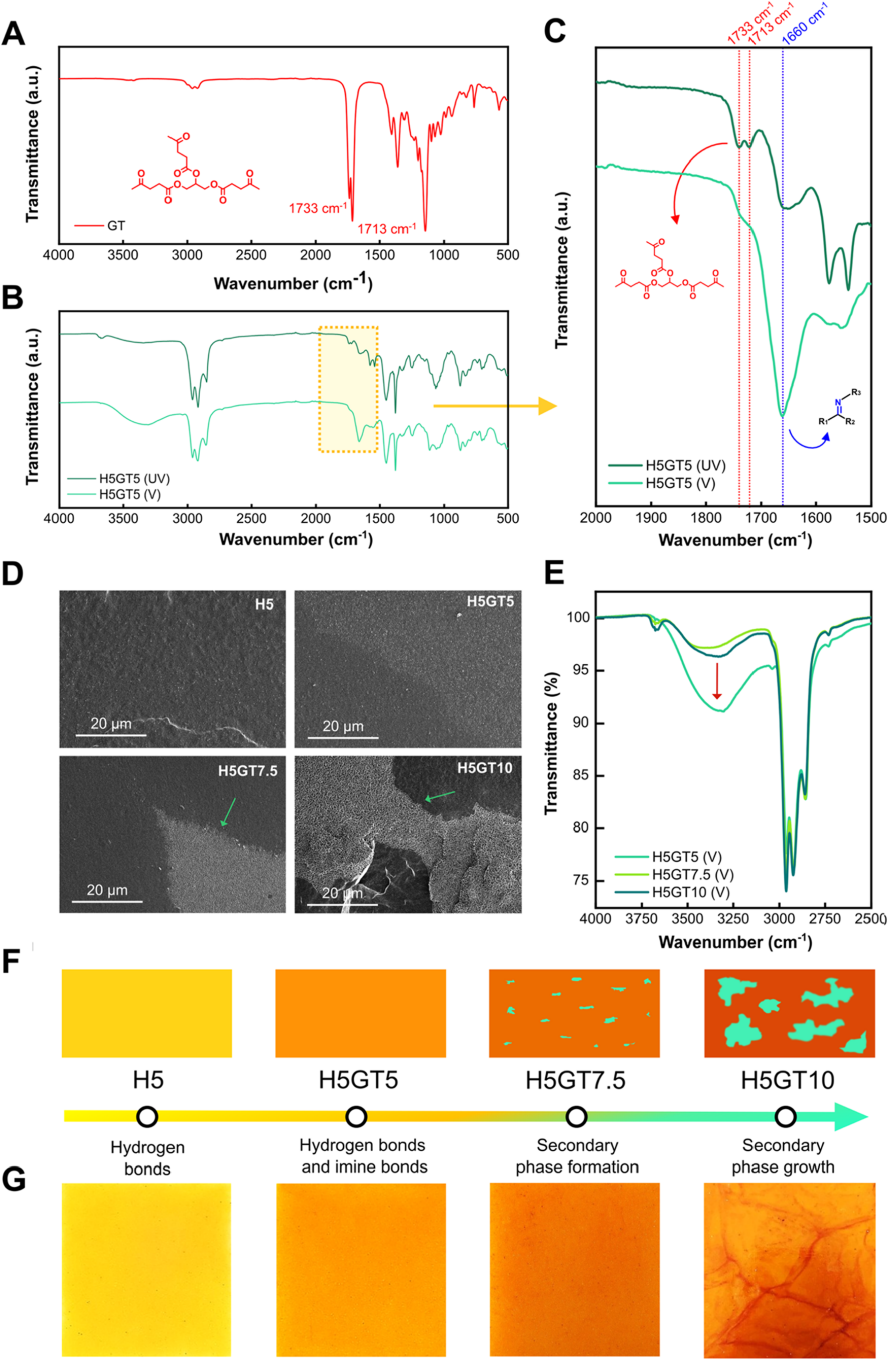
FTIR spectra of (A) GT, (B) H5GT5 formulation before (UV) and after vulcanization process (V). (C) Highlight of H5GT5 FTIR spectra showing the characteristic peaks of GT (red) and imine bond (blue). (D) SEM images of the vulcanized rubber formulations, the green arrows show the secondary phase formed by the reaction between GT and HMDA. (E) Comparison of the FTIR spectra of the vulcanized compounds, showing the intensity variation of the peak ascribable to the stretching of the hydroxyl groups. (F) Graphical representation of the formation of the secondary phase (green spots) related to the increase of the GT content. (G) Optical photographs of the vulcanized samples (110 mm × 110 mm) showing the formation of the secondary phase.

### Assessing the ENR Compounds Recyclability

Once the presence of imine bonds confirmed the formation of a potentially reversible network, the four ENR compounds underwent the recycling protocol. As detailed in the Experimental section and shown in Figure 3A, the virgin samples were initially cut into smaller pieces, then ground into powder through cryomilling. The resulting powder was compacted using a two-roll mill, followed by reprocessing in a mold using a hot press to produce square-shaped samples for further evaluation of the material properties, thereby assessing the efficiency of the recycling protocol.

**Figure 3:**
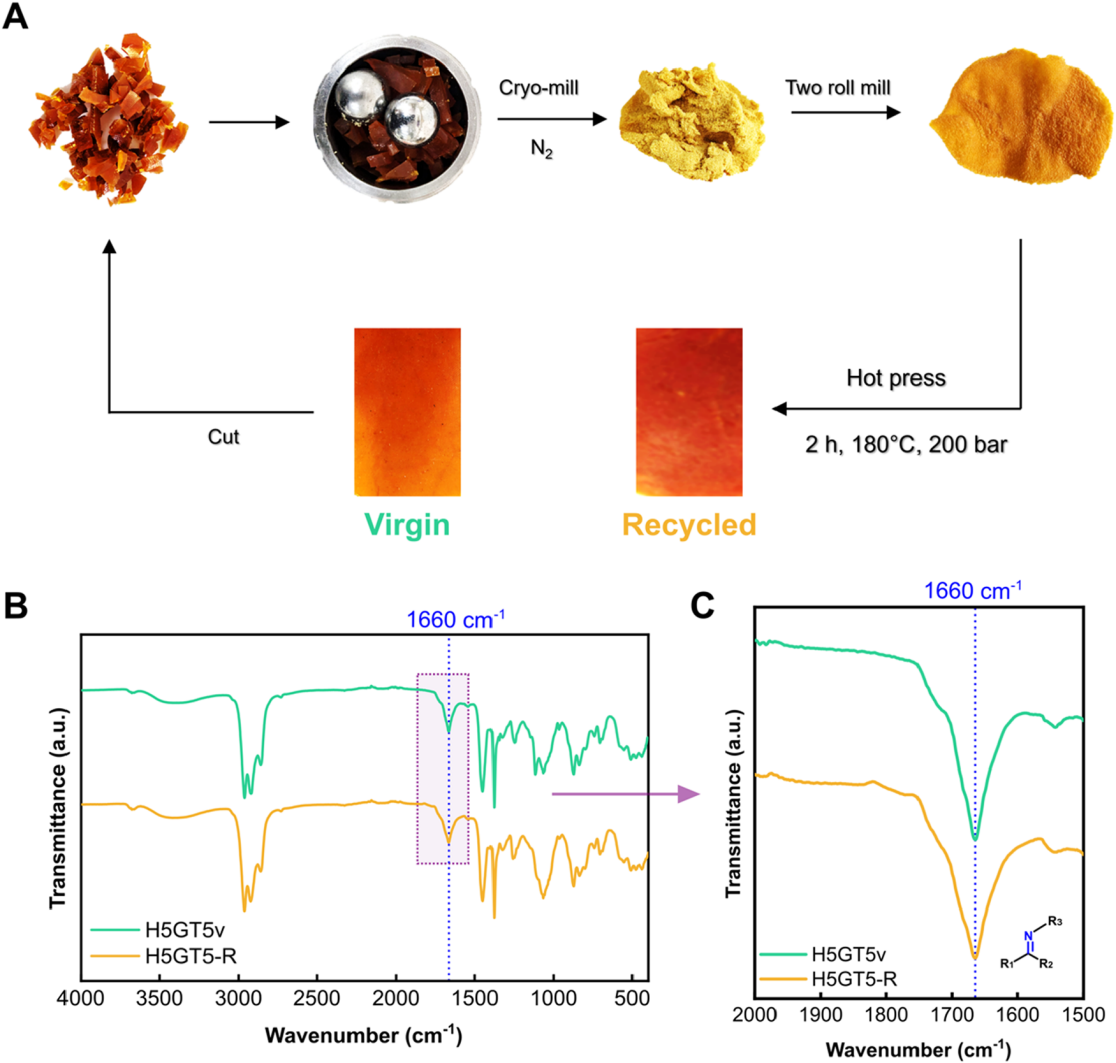
(A) Recycling protocol scheme. (B) Comparison of the FTIR spectra of H5GT5 virgin (v) and recycled (-R) compounds. (C) Highlight of the FTIR spectra in the range 2000 – 1500 cm^−1^, showing the peak ascribed to imine bond (blue dashed line).

FTIR analysis was employed to observe potential changes in the chemistry of the system after recycling. In the FTIR spectra shown in Figure 3B, the recycled sample H5GT5-R exhibited the same characteristic peaks as the virgin material (H5GT5v). Specifically, both displayed the hydroxyl stretching between 3600 and 3200 cm^–1^, as well as the distinctive peak at 1660 cm^–1^ (as shown in the inset of Figure 3C), attributed to imine bonds, confirming the successful reformation of the reversible covalent bonds after the recycling protocol.

Interestingly, the H5 virgin and recycled samples (Figure S5A) exhibited an increase in the broad peak between 3600 and 3200 cm^–1^, likely due to unreacted functionalities of HMDA in the virgin sample, which were subsequently consumed after the second heating treatment, generating more hydroxyl groups within the system. The spectra of the compounds with 7.5 and 10 phr of GT, shown in Figure S5B,C respectively, confirmed the presence of imine bonds after recycling. The potential structural changes after recycling were assessed by evaluating the ρ_crosslink_ of both virgin and recycled compounds using via swelling test in toluene, using the Flory–Rehner method^39,40^ to relate swelling behavior to network cross-linking, according to eq 1. As shown in Figure 4A, for the virgin samples ρ_crosslink_ increased with GT content, evidencing that the addition of the biobased molecule actively contributed to the formation of a denser network (results also reported in Table S1). The reference sample H5 exhibited an increase in ρ_crosslink_ after recycling, further suggesting that during the first vulcanization step not all amine functionalities reacted with ENR’s epoxy groups and thus, further cross-linking occurred upon reprocessing (consistent with the changes observed in the FTIR spectra in Figure S5A). On the contrary, H5GT5 showed nearly identical ρ_crosslink_ values before and after recycling. Thus, the hybrid network was efficiently reformed during the recycling process, with no major integrity loss. H5GT7.5 showed a higher ρ_crosslink_ after recycling compared to its virgin counterpart. Similar results were reported by Utrera-Barrios et al.^58^ in carboxylated nitrile rubber (XNBR) showing that ρ_crosslink_ increased with recycling cycles due to progressive structural rearrangements. In contrast, H5GT10-R exhibited a reduction in ρ_crosslink_ compared to virgin material, suggesting that, at high GT contents, the network may have undergone thermomechanical degradation during the recycling process, leading to a loss in cross-linked structure and potentially compromising recyclability.

**Figure 4:**
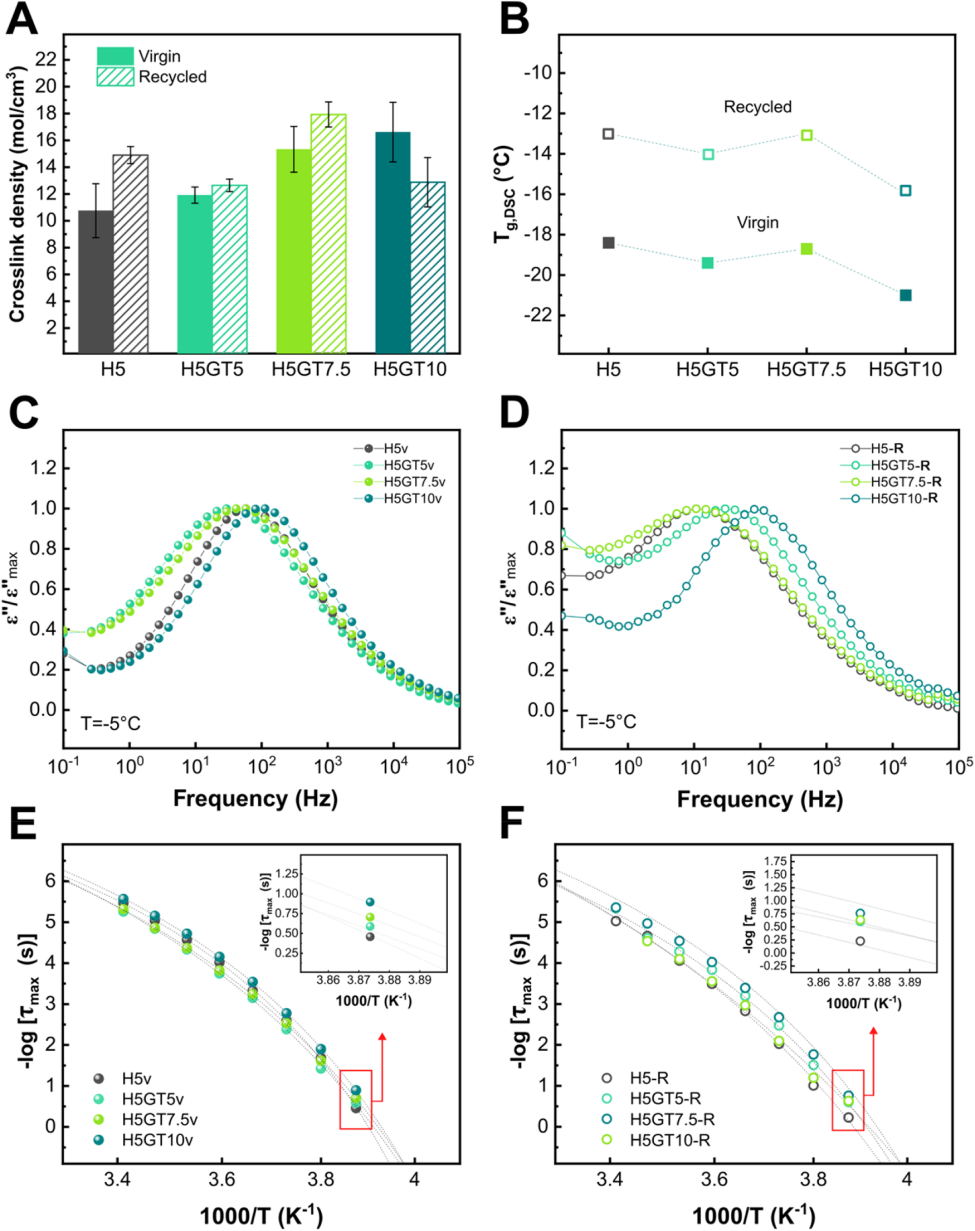
(A) ρ_crosslink_ of the virgin (solid) and recycled (hatched) samples. (B) Glass transition temperature (*T*_g,DSC_) extrapolated from DSC of virgin (solid) and recycled (hollow) rubber compounds. Normalized dielectric loss (ε″/ε′′_max_) of (C) virgin and (D) recycled compounds. Activation plots of virgin (E) and recycled (F) compounds, showing the variation of – log­(τ_max_) as a function of 1000/*T*, fitted using the Vogel–Fulcher–Tammann (VFT) equation (eq 5). Insets of Figure 4E and 4F highlight – log­(τ_max_) values at a selected temperature of 258 K (corresponding at approximately – 15 °C).

Further DSC investigations demonstrated that up to 7.5 phr of GT, the *T*_g_ of the virgin compounds remained largely unchanged compared to H5v (thermograms and corresponding values are reported in Figure S6 and Table S1, respectively). As shown in Figure 4B, H5v, H5GT5v and H5GT7.5v exhibited similar *T*_g_ values in the order of −18 °C, showing that the incorporation of GT, even at relatively high concentrations, did not significantly affect the rubber chain mobility, although the networks formed were more densely cross-linked. However, in H5GT10v, *T*_g_ decreased to −21 °C, showing a softening effect due to the excess GT. These results did not align well with the H5GT10v ρ_crosslink_ values discussed earlier, as one would typically expect a decrease in mobility with an increase in ρ_crosslink_. However, the presence of a secondary phase may be acting as an auxiliary network consisting of additional GT-HMDA linkages. It can then be hypothesized that the observed ρ_crosslink_ values were originated from two distinct contributions: the first, from the cross-linked network formed during vulcanization with covalent bonds between ENR and HMDA and hydrogen bonding, and the second from the dynamic GT-HDMA imine linkages, which arise due to an excess of GT. Previous study by Blume et al.^59^ suggests that incorporating a liquid polymer (e.g., plasticizer) into polybutadiene rubber (BR) reduces the average molecular weight, thereby hindering the ideal formation of cross-links. However, in this case, the secondary phase also creates an additional network, which increases ρ_crosslink_ evidencing the contribution of intramolecular cross-links and trapped entanglements, as schematically represented in Figure 2F.

Following recycling, all compounds showed an increase in *T*_g_, which can be attributed to the reorganization of the dynamic network during reprocessing (Figure 4B). The activation of imine metathesis and potential transamination^21^ likely promoted the formation of more densely cross-linked structures, resulting in higher *T*_g_.

BDS analysis was conducted to provide further evidence of changes in the structure and dynamics of the rubber compounds, considering both GT content and virgin versus recycled conditions. BDS measurements were carried out in the frequency range 10^–1^–10^6^ Hz across variable temperatures, allowing the analysis of dipolar relaxation processes in the rubber matrix, with particular focus on the temperature range where a frequency maximum was observed. This maximum represents evidence of the α-relaxation, which is associated with the segmental motions of the rubber chains in the glass transition region. The progressive shift of the dielectric loss maxima to higher frequencies with increasing temperature, characteristic of a thermally activated process like glass transition, was clearly observed in the dielectric loss profiles of both virgin and recycled compounds (Figure S7). The dielectric loss spectra shown in Figure 4C,4D for the virgin and recycled compounds, respectively, were measured within the available frequency range at a selected temperature (T = −5 °C), where they exhibited a well-resolved and centered peak. The complex dielectric permittivity ε*­(ω), composed of its real (ε′) and imaginary (ε″) parts, was analyzed as defined in eq 2. To facilitate comparison and account for the nonabsolute nature of the dielectric loss measurements, the data were normalized to the maximum value of each curve.

As shown in Figure 4C, both virgin H5GT5 and H5GT7.5 showed a shift of the dielectric loss peak toward lower frequencies compared to the reference (H5), indicating that these systems are more rigid. This behavior was consistent with cross-link density measurements, which also suggested a more tightly cross-linked network. In contrast, H5GT10 exhibited a shift toward higher frequencies, likely due to the plasticizing effect of GT, which loosened the molecular packing of the rubber chain segments and increased molecular mobility.^60^ After recycling (Figure 5D), the general trend observed for the virgin samples was still observed, with H5GT10 showing the least restricted dynamics, consistent with the lower *T*_g_ observed in the DSC results. A more detailed comparison between virgin and recycled compounds (Figure S8) revealed that reprocessing restricts the mobility of the rubber chains, as indicated by the shift of the loss peak to lower frequencies. Although a secondary phase was detected in some compounds, no secondary relaxation process was observed in the dielectric spectra. This was likely because the transition associated with the secondary phase occurred within the same frequency and temperature range as the primary α-relaxation of the rubber matrix, making it indistinguishable.^61^ This hypothesis was further supported by DSC analysis (Figure S6), where no distinct secondary glass transition was detected. To extract quantitative parameters describing the relaxation processes, the dielectric loss spectra were fitted using the Havriliak–Negami (HN) model,^42^ as reported in eq 3. The calculated parameters were used to create the activation plots shown in Figure 4E for the virgin samples and in Figure 4F for the recycled ones. The plots display the variation of the maximum relaxation time τ_max_ (defined by eq 4) expressed as −log­(τ_max_) with 1000/T, following a Vogel–Fulcher–Tammann–Hesse (VFTH, calculated by eq 5) behavior typically associated with cooperative segmental dynamics in polymers.^43^ For the virgin materials, a clear dependence on GT content is observed. Specifically, the reference H5 compound exhibited the fastest dynamics. As shown in the inset of Figure 4E, upon introducing GT, a progressive shift toward slower relaxation (higher τ_max_) was recorded for H5GT5, H5GT7.5 and H5GT10 indicating reduced chain mobility. Upon recycling (Figure 4F), the materials displayed relaxation times that are generally different from those of their virgin counterparts. However, as visible in the inset of Figure 4F, H5GT5 exhibited a relaxation profile in the recycled state that is remarkably similar to that of the virgin material, further confirming that the dynamic network is effectively reformed.

**Figure 5:**
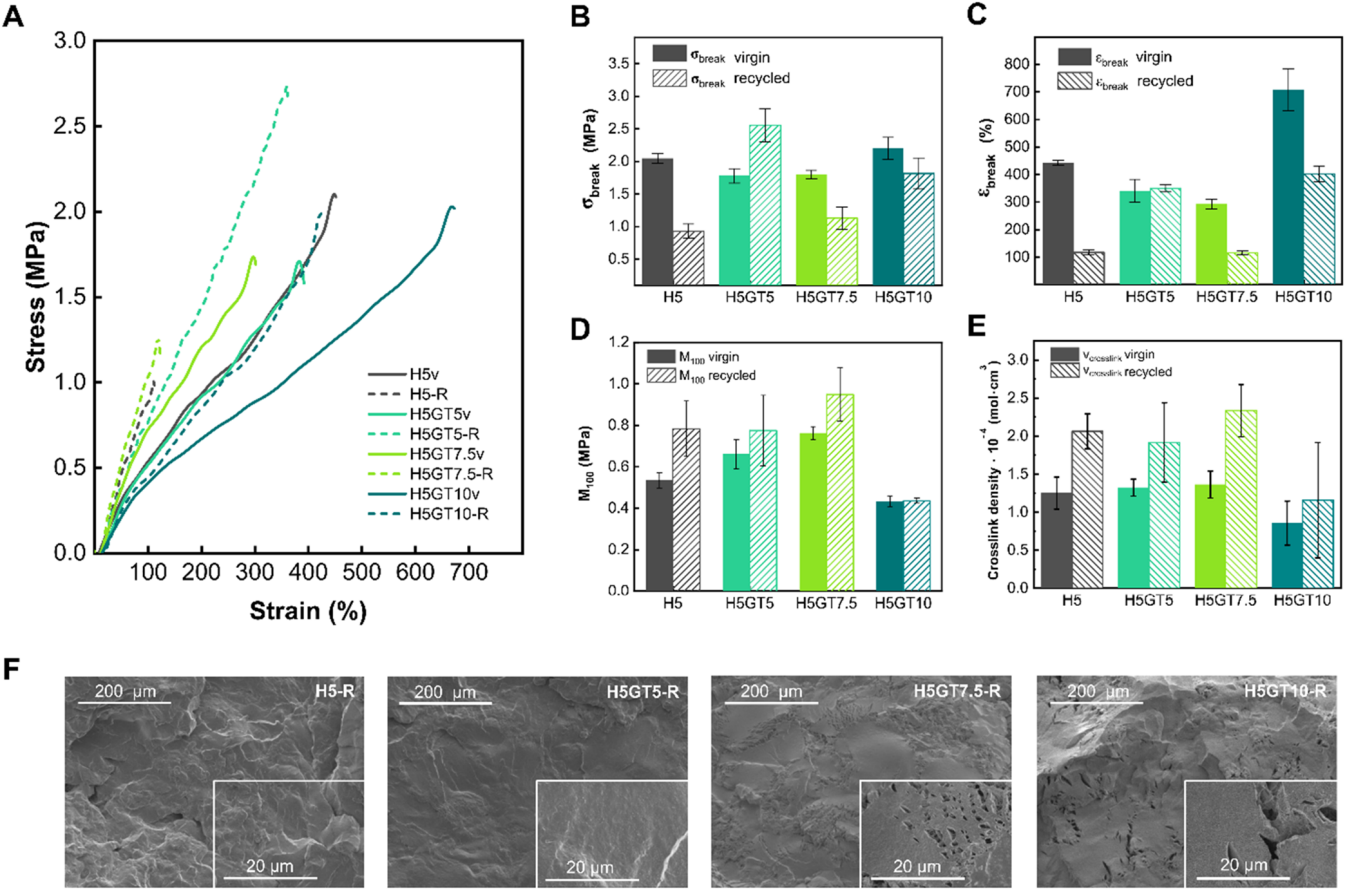
(A) Representative stress–strain curves for the virgin (v, solid line) and recycled (-R, dashed line) compounds. Trends of (B) σ_break,_ (C) ε_break_, (D) M_100_ and (E) ν_crosslink_ of the virgin (v, solid) and recycled (-R, hatched) ENR compounds, respectively. (F) SEM micrographs (secondary electron mode) of the recycled compounds. Insets show higher magnification images.

The mechanical behavior of the rubber compounds was evaluated through tensile testing, aiming not only to assess the performance of the newly developed ENR–HMDA–GT systems, but also to determine the effectiveness of the recycling process by verifying the recovery of mechanical properties after reprocessing. Dog bone specimens were tested with tensile test to assess stress–strain behavior of the virgin and recycled materials, with *M*_100_, σ_break_ and ε_break_ used as key indicators of material stiffness, strength and flexibility. Representative stress–strain curves are presented in Figure 5A, while the full results of the tensile tests are summarized in Table S2. The effect of GT on the mechanical properties can be closely linked to the cross-link density and molecular dynamics trends reported in Figure 4A and 4C. For the virgin compounds up to 7.5 phr, the increasing GT content led to a denser cross-linked network, which limited chain mobility and resulted in a slight decrease in both σ_break_ and ε_break_. Notably, the mechanical performances were comparable^62^ or even superior^63^ to other fully biobased ENR systems vulcanized with sustainable cross-linking agents reported in the literature. As shown in Figure 5B, σ_break_ went from 2.04 MPa for H5v to 1.78 and 1.80 MPa for H5GT5v and H5GT7.5v, respectively. Similarly, as reported in Figure 5C, the ε_break_ decreased from 444% for H5v, to 341% for H5GT5v and further to 293% for H5GT7.5v. This behavior suggested that GT limited the flexibility of the material and weakened its ability to withstand high stresses. However, at 10 phr GT, a different behavior was observed. H5GT10v exhibited a σ_break_ increase to 2.20 MPa, deviating from the previous decreasing trend, while ε_break_ drastically increased to 708%, indicating a significant improvement in flexibility. This confirmed that, at such concentrations, GT introduced a plasticizing effect, redistributing stress more effectively and enhancing the material’s flexibility and strength.

Upon recycling, significant differences were observed among the rubber compounds, reflecting variations in their ability to recover mechanical properties. H5-R exhibited the lowest recyclability, with substantial reductions in both σ_break_ and ε_break_ (Figure 5B,5C, respectively), suggesting that only hydrogen bonds cannot restore an effective cross-linked network after reprocessing. Furthermore, the degradation of ENR-HDMA covalent bonds within the rubber matrix likely occurred, contributing to the observed mechanical performance drop. Among the rubber systems embedding the combined reversible cross-link network, H5GT7.5-R and H5GT10-R show partial recovery of mechanical properties, though they did not fully regain the performance of their virgin counterparts. The presence of the secondary phase, rich in imine bonds (Figure S4), might have compromised the overall material integrity. The imine bonds were likely concentrated in the secondary phase and not in the rubber phase, resulting in regions lacking covalent reversible bonds. As a result, the rubber phase exhibited behavior similar to H5-R, where the absence of sufficient reversible covalent bonds hindered the formation of an effective CAN.

A notable result was observed for H5GT5-R, which exhibited a significant increase in σ_break_, which raised from 1.78 MPa in the virgin state to 2.56 MPa after recycling (Figure 5B), without any loss in ε_break_ (Figure 5C). This strongly indicates that this compound achieved an optimal balance between covalent and noncovalent interactions, enabling more effective network reconstruction after reprocessing.

As shown in Figure 5D, *M*_100_ increased with GT content up to 7.5 phr in the virgin samples, indicating a progressive stiffening of the material due to the formation of a denser and more constrained network (Table S2), which supports the reduced chain mobility observed by the mechanical behavior and swelling tests. Upon recycling, all samples up to 7.5 phr of GT showed a further increase in *M*_100_, confirming that the recycled compounds became more rigid. The enhanced stiffness was particularly evident for H5-R, H5GT5-R, and H5GT7.5-R, which supports the idea that reprocessing promoted additional physical interactions and morphological rearrangements within the network. In contrast, H5GT10-R maintained a lower modulus, likely due to the extensive formation of the secondary phase, which resulted in a more heterogeneous material with a distinct mechanical behavior compared to the other formulations.

To further assess the network architecture of the rubber compounds, ν_crosslink_ was estimated using the Mooney–Rivlin model based on uniaxial tensile data. This analysis offers a complementary perspective to the swelling-based Flory–Rehner method discussed earlier, focusing primarily on the contribution of chemical cross-links to the material’s elastic response. The values of the ν_crosslink_, calculated with eq 7, are summarized in Table S3. As shown in Figure 5E, the ν_crosslink_ values in the virgin samples showed a steady increase with GT content up to 7.5 phr, confirming that the additive actively participated in network formation by reacting with HMDA, consistent with the swelling results. For H5GT10, the Mooney-derived ν_crosslink_ dropped, further suggesting that at high GT concentrations, the molecule may no longer effectively participate in network formation within the rubber, because likely consumed in the formation of the secondary phase, which had minimal contribution to the elastic response of the material. Upon recycling, an increase in ν_cross‑link_ was observed for all systems, suggesting that the reprocessing step promoted a more compact and physically constrained network. This effect may derive from phase rearrangements and the densification or reorganization of existing hydrogen bonds, which enhanced their contribution to the elastic response of the material.^64^ Unlike permanent covalent cross-links, these physical interactions were not detected by swelling tests, while significantly affecting the mechanical properties of the recycled formulations.

The morphological changes in the compounds after the recycling process were investigated by SEM imaging and presented in Figure 5F. As expected, H5-R exhibited evident cracks and voids, which are consistent with the poor mechanical performance reported in Figure 5B,5C. Both H5GT7.5-R and H5GT10-R also exhibited a considerable number of cracks, indicating that macroscale fragments of the recycled powder did not properly coalesce during reprocessing leading to a fragmented final microstructure. This could be attributed to the insufficient presence of imine bonds within the rubber phase (Figure S4), which inhibited the formation of a reversible cross-linked network and may have contributed to material degradation during recycling. In contrast, H5GT5-R exhibited a smooth surface closely resembling that of its virgin counterpart (Figure 2D), thus indicating the material’s ability to fully re-establish a cohesive and continuous phase upon recycling. This morphological integrity, combined with the recovered mechanical properties, confirmed that the balanced ratio of GT and HMDA in this formulation (5 phr GT and HMDA) successfully enabled the formation of an efficient dynamic network.

## Conclusions

In this study we successfully developed a biobased epoxidized natural rubber network using glycerol trilevulinate (GT) and hexamethylene diamine (HMDA) as cross-linking agents. This innovative system incorporates reversible covalent (imine) and noncovalent (hydrogen) bonds, which enhance both recyclability and mechanical stability. Comprehensive characterization confirmed the formation of a covalent adaptive network, demonstrating the robustness and flexibility of the system. Mechanical testing showed that the H5GT5 compound maintained its properties after recycling, with a significant increase in σ_break_ from 1.78 to 2.56 MPa, while retaining a similar ε_break_ compared to its virgin counterpart (∼340%). FTIR, DSC and BDS analyses confirmed the dynamic exchange of imine bonds and the formation of a stable, yet structurally distinct network. The optimal balance between covalent and noncovalent interactions allowed for an effective reprocessing without significant performance loss. In contrast, H5GT7.5 and H5GT10 exhibited limited recyclability in terms of mechanical properties, likely due to phase separation, which hindered the reformation of the cross-linked network, as shown by scanning electron microscopy investigations. These results position H5GT5 as a promising candidate for the development of fully biobased, recyclable rubbers with consistent mechanical properties postrecycling. Future efforts could focus on better understanding the reaction mechanisms governing network formation and reformation, enabling more precise control over recyclability and material performance. Optimizing vulcanization and exploring dynamic-compatible fillers may further enhance the versatility of this ketone–amine approach, which could also be extended beyond rubber systems to other recyclable polymer networks.

## Supplementary Material



## Notes

**Figure S1**: Isothermal DSC thermogram of, used to estimate the curing time; **Figure S2**: FTIR spectra of representative compounds before and after vulcanization**; Figure S3**: SEM micrographs showing the presence of a secondary phase**; Figure S4**: Proposed side reaction mechanism between GT and HMDA forming imine bonds**; Figure S5**: FTIR spectra comparing virgin and recycled compounds**; Table S1**: Values of cross-link density from swelling tests, glass transition temperatures from DSC**; Figure S6**: DSC thermograms of virgin and recycled compounds with *T*_g_ extrapolation**; Figure S7**: Dielectric loss (ε″) vs frequency at various temperatures**; Figure S8**: Normalized dielectric loss (ε″/ ε″_max_) comparison of virgin and recycled compounds**; Table S2**: Mechanical properties of the virgin and recycled compounds**; Table S3**: C1, C2 values and Mooney-Rivlin cross-link densities derived from tensile data (PDF)
