# Supplementary material for: From Levulinic Acid to Imines: Creating Biobased, Recyclable, Cross-linked Rubbers through Covalent Adaptive Networks
Source: ACS Polym Au. 2025 Sep 26;5(5):656–68. doi: 10.1021/acspolymersau.5c00108 (PMC12511985; doi:10.1021/acspolymersau.5c00108)
Supplement: Supplementary file 1 [file lg5c00108_si_001.pdf]

SUPPORTING INFORMATION

From Levulinic Acid to Imines: Creating Bio-based, Recyclable, Crosslinked Rubbers through Covalent Adaptive Networks

Luca Lenzi[a,b], Juan Carlos Chicharro[c], Micaela Degli Esposti[a,b], Davide Morselli[a,b,*], Marianella Hernández Santana[c,*], Paola Fabbri[a,b]

[a] *Department of Civil, Chemical, Environmental and Materials Engineering (DICAM), Università di Bologna, Via Terracini 28, 40131 Bologna, Italy*

[b] *National Interuniversity Consortium of Materials Science and Technology (INSTM), Via Giusti 9, 50121 Firenze, Italy*

[c] *Institute of Polymer Science and Technology (ICTP), CSIC, Juan de La Cierva 3, 28006 Madrid, Spain*

Corresponding authors: marherna@ictp.csic.es; davide.morselli6@unibo.it

Contents **Page**

Figure S1 .... S2
Figure S2 .... S3
Figure S3 .... S4
Figure S4 .... S4
Figure S6 .... S5
Table S1 .... S6
Figure S6 .... S6
Figure S7 .... S7
Figure S8 .... S8
Table S2 .... S8
Table S3 .... S9

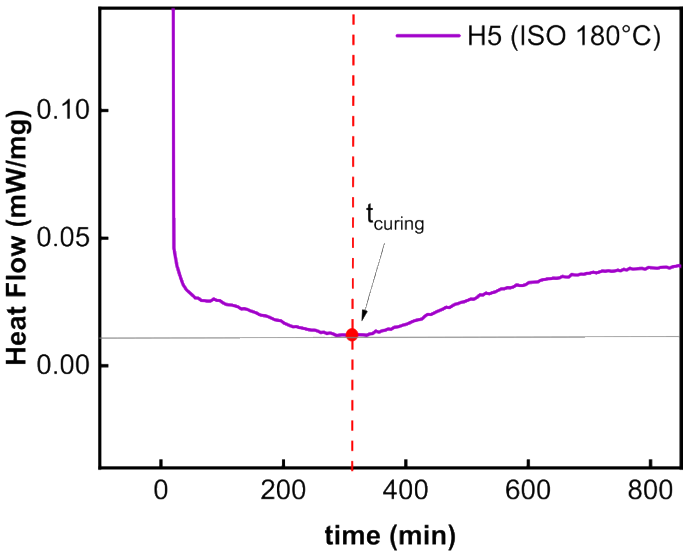

Figure S1. Isothermal DSC thermogram obtained analysing the H5 sample at 180 °C. The intersection between the DSC curve (violet) and the red dashed line indicates the estimated curing time (approx. 265 min).

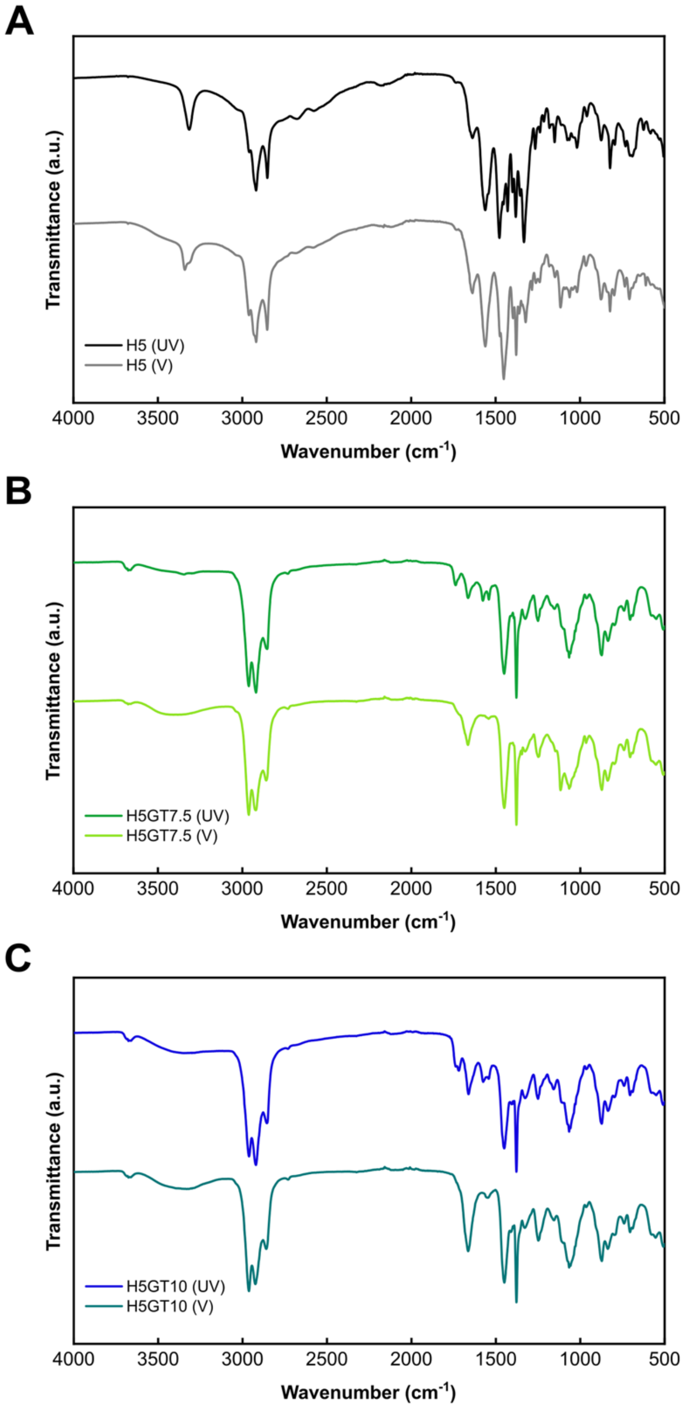

Figure S2. FTIR spectra of the studied compounds before (UV) and after vulcanization (V). (A) H5, (B) H5GT7.5 and (C) H5GT10 compounds, respectively.

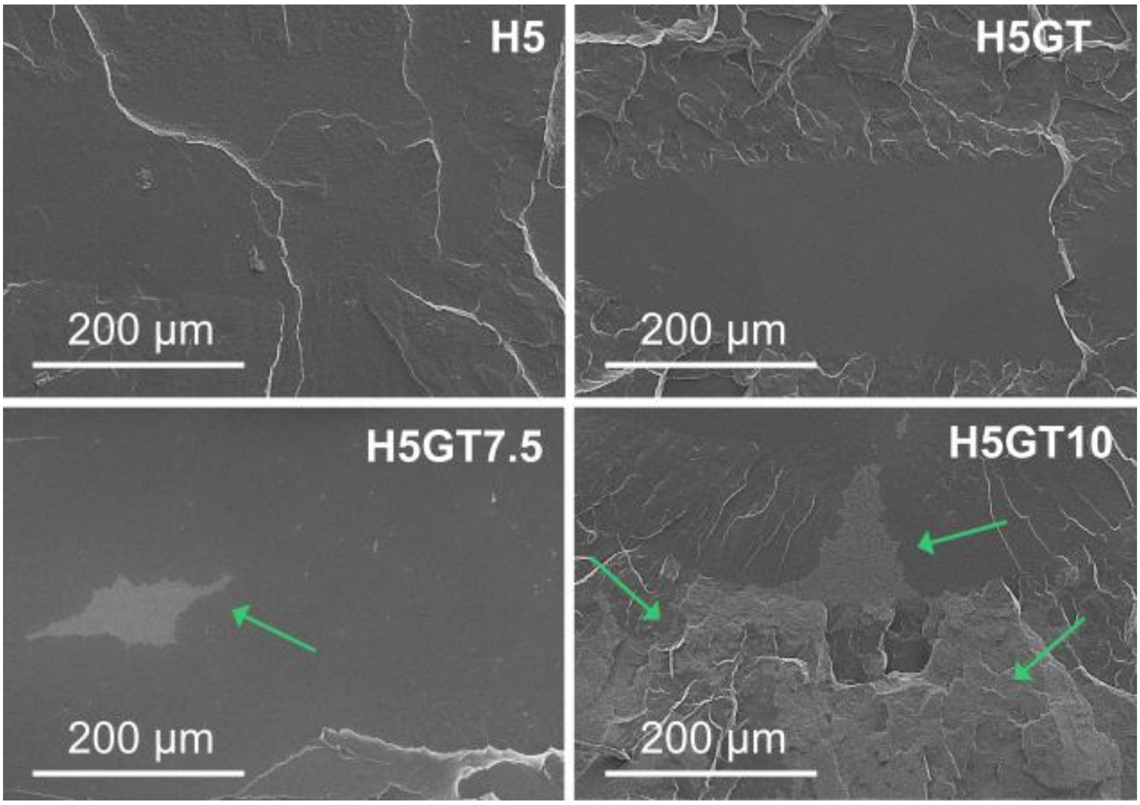

Figure S3. SEM micrographs of the virgin samples. The green arrows indicate the presence of a secondary phase within the rubber matrix.

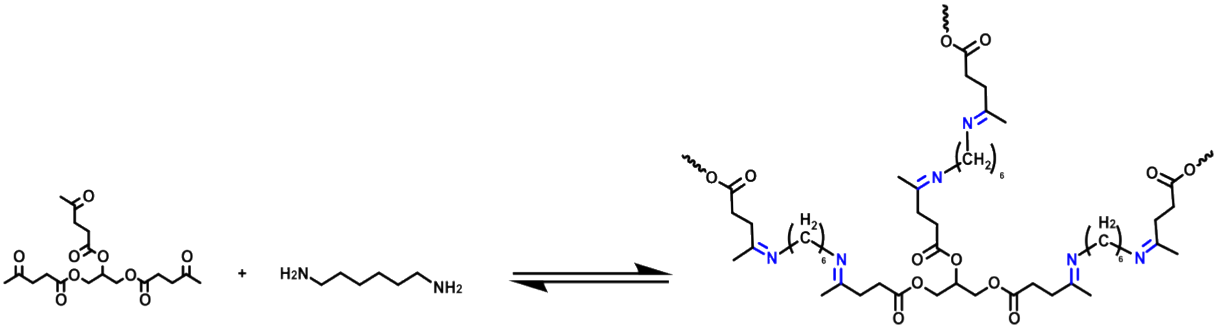

Figure S4. Possible side-reaction between GT and HMDA that could occur in the H5GT7.5 and H5GT10 compounds. Imine bonds formed by the reaction of GT's ketones and HMDA's amine groups are reported in blue.

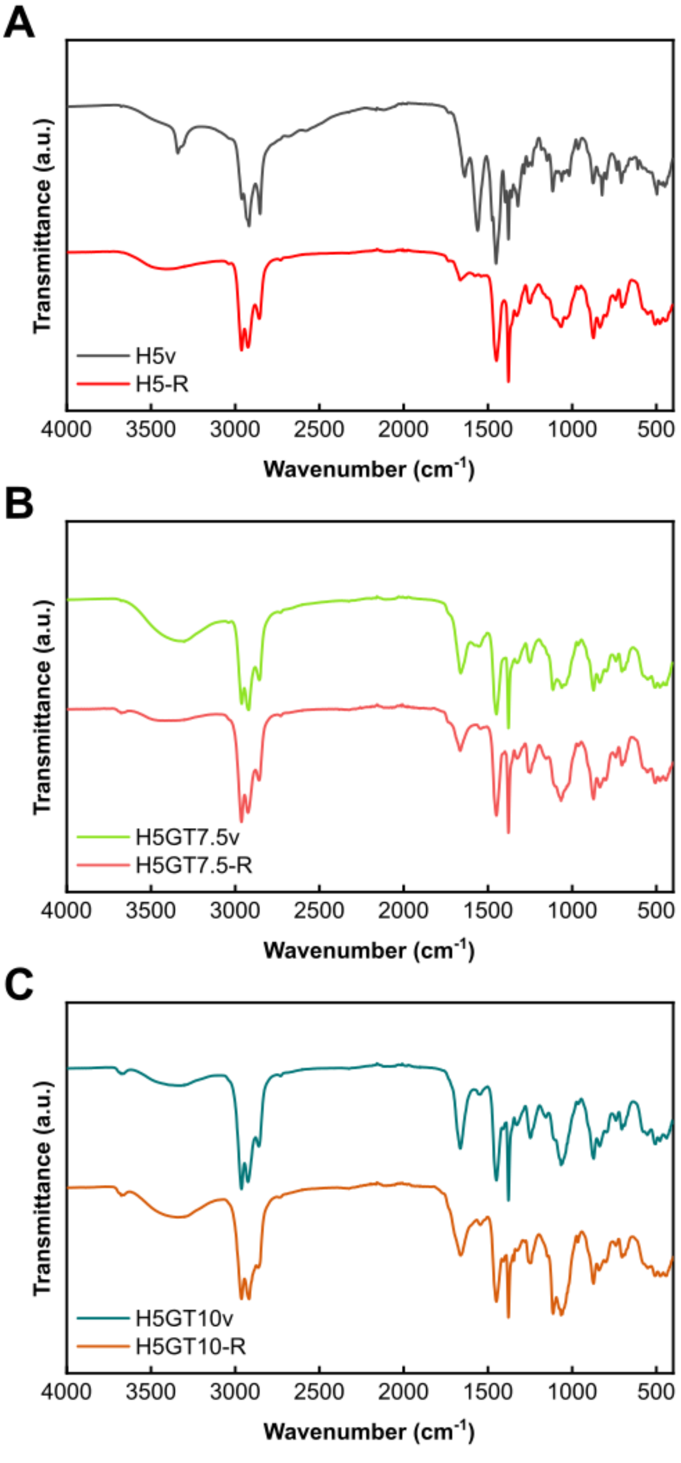

Figure S5. FTIR spectra of the compounds pre (v) and post (-R) recycling protocol. (A) H5, (B) H5GT7.5 and (C) H5GT10 compounds, respectively.

Table S1. Crosslink density ($\rho_{crosslink}$), glass transition temperature from DSC analysis ($T_{g,DSC}$) of the virgin (v) and recycled (-R) samples.

**Sample**	**$\rho_{crosslink}$ ·10^{-5} (mol·cm^{-3})**	**$T_{g,\ DSC}$ (°C)**
H5v	10.8 ± 2.0	-18
H5GT5v	11.9 ± 0.6	-19
H5GT7.5v	15.3 ± 1.7	-19
H5GT10v	16.6 ± 2.2	-21
H5-R	14.9 ± 0.6	-13
H5GT5-R	12.6 ± 0.5	-14
H5GT7.5-R	17.9 ± 0.9	-13
H5GT10-R	12.9 ± 1.8	-16

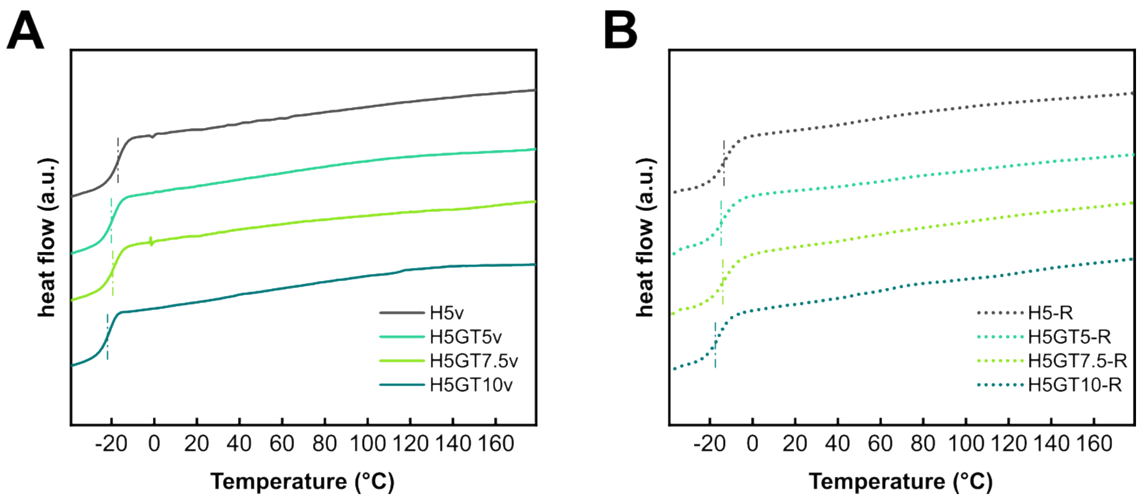

Figure S6. DSC thermograms of (A) virgin (solid line) and (B) recycled (dotted line) compounds heated at 20°C·min^{-1}. The vertical dashed lines indicate the point where the glass transition temperatures were extrapolated.

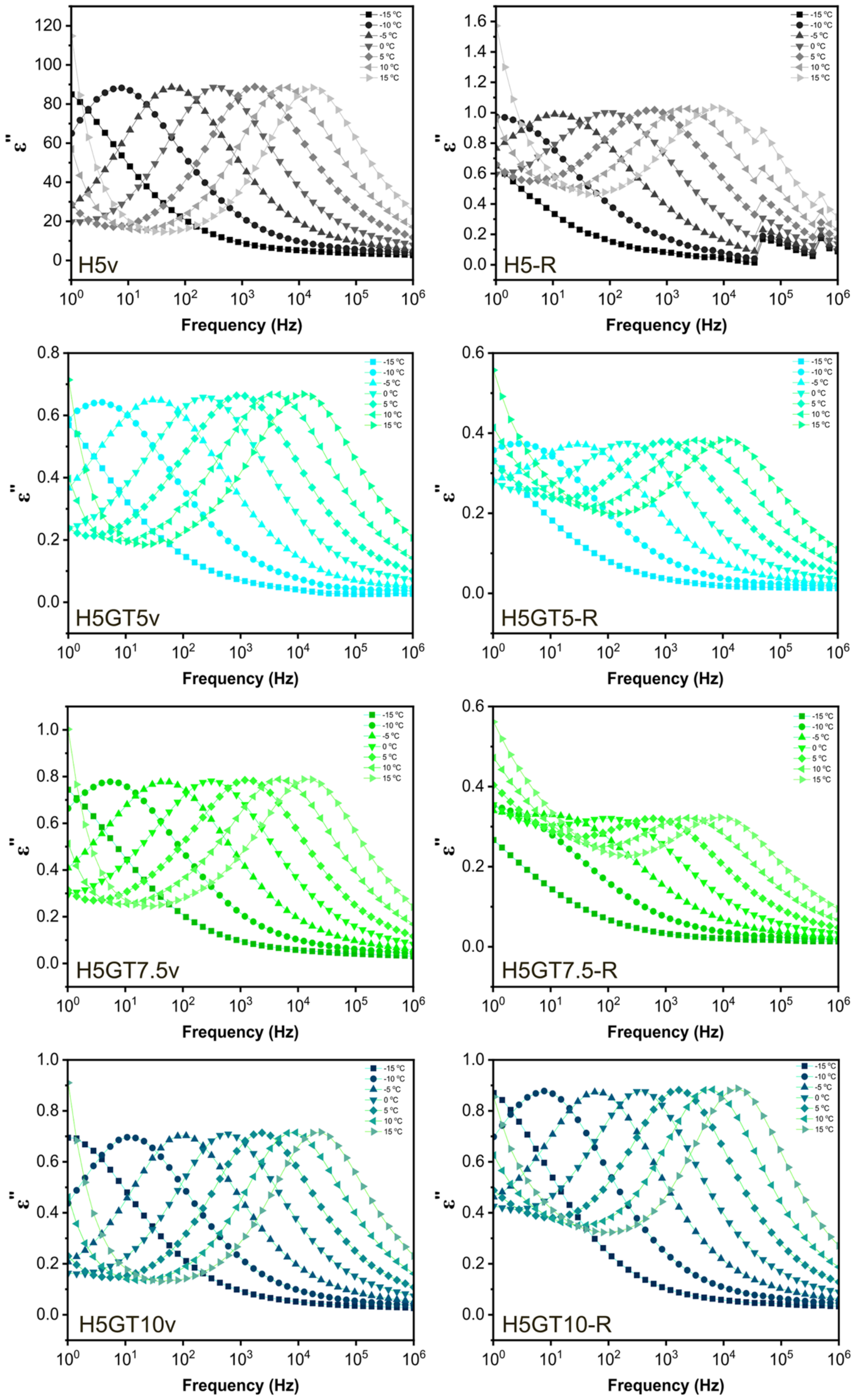

Figure S7. Dielectric loss (ε′′) of the studied rubber compounds as a function of broad frequency range evaluated at different temperatures.

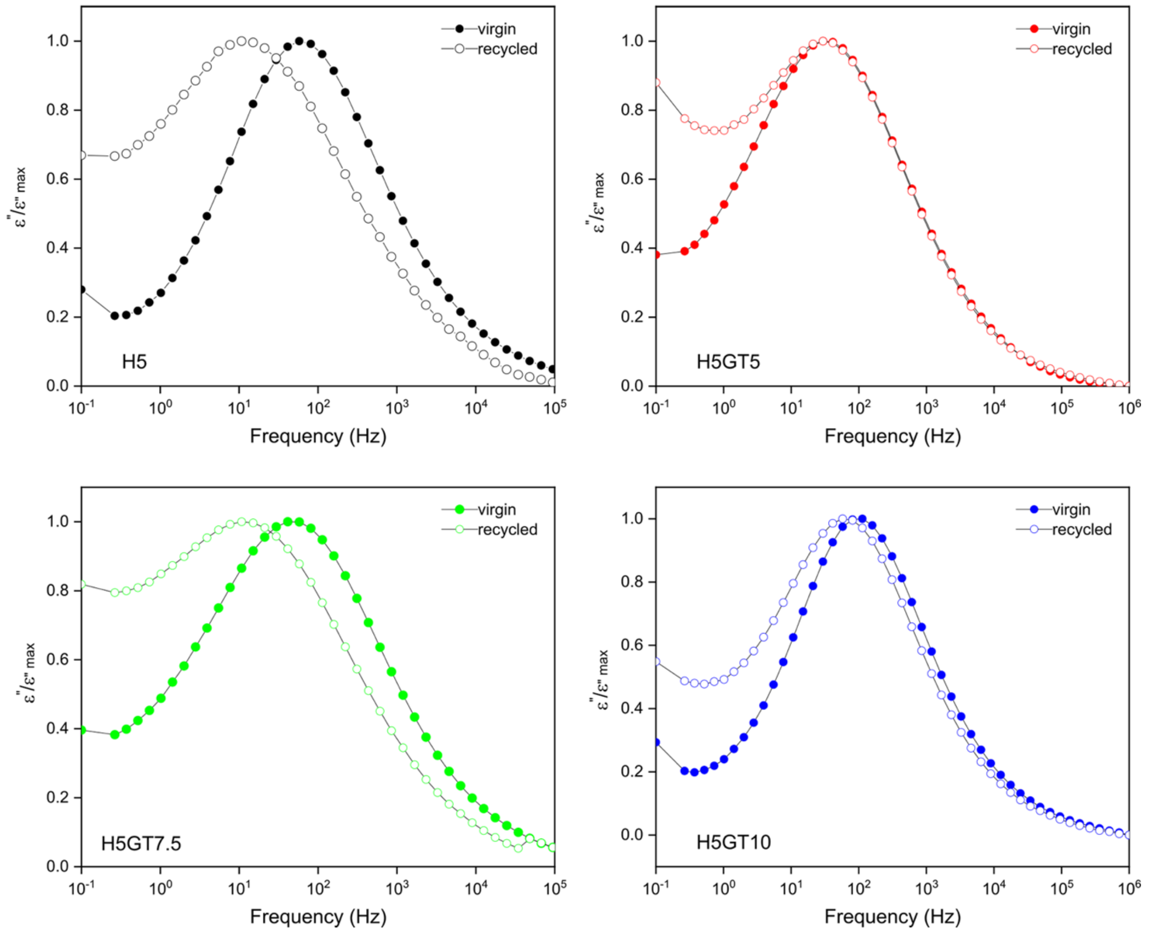

Figure S8. Normalized dielectric loss ($\varepsilon''/\varepsilon''_{max}$) of virgin (solid dot) and recycled (hollow dot) rubber compounds as a function of the frequency.

Table S2. Modulus at 100% of strain (M_{100}), Stress at break (σ_{break}), elongation at break (ε_{break}) derived from tensile tests of the virgin (v) and recycled (-R) compounds.

Sample	M_{100} (MPa)	σ_{break} (MPa)	ε_{break} (%)
H5v	0.53 ± 0.04	2.04 ± 0.07	444 ± 9
H5GT5v	0.66 ± 0.17	1.78 ± 0.11	341 ± 42
H5GT7.5v	0.76 ± 0.03	1.80 ± 0.07	293 ± 18
H5GT10v	0.43 ± 0.02	2.20 ± 0.27	708 ± 76
H5-R	0.78 ± 0.13	0.93 ± 0.11	118 ± 10
H5GT5-R	0.78 ± 0.01	2.56 ± 0.25	351 ±13
H5GT7.5-R	0.95 ± 0.13	1.13 ± 0.17	117 ± 6
H5GT10-R	0.44 ± 0.01	1.82 ± 0.24	403 ± 28

Table S3. C1 and C2 factor of extrapolated from the linear fitting of the stress and strain curves, and the relative Mooney-Rivlin crosslink density ($v_{crosslink}$) values of the virgin (v) and recycled (-R) compounds calculated using Eq. 7.

**Formulation**	**C1**	**C2**	**$v_{crosslink}$ · 10^{-4} (mol·cm^3)**
H5v	15.49 ± 0.12	1.48 ± 0.21	1.25± 0.21
H5GT5v	16.39 ± 0.09	5.29 ± 0.17	1.32 ± 0.23
H5GT7.5v	16.88 ± 0.24	10.48 ± 1.72	1.36± 0.11
H5GT10v	10.59 ± 0.12	4.62 ± 0.30	0.85 ± 1.02
H5-R	25.56 ± 1.66	5.16 ± 2.81	2.06 ± 1.02
H5GT5-R	23.74 ± 0.36	1.82 ± 0.65	1.92 ± 0.35
H5GT7.5-R	28.91 ± 1.13	4.57 ± 1.72	2.33 ± 0.34
H5GT10-R	14.33 ± 0.17	5.05 ± 0.28	1.16 ± 0.76
